# Cortical cerebral microinfarcts on 7T MRI: Risk factors, neuroimaging correlates and cognitive functioning – The Medea-7T study

**DOI:** 10.1177/0271678X211025447

**Published:** 2021-06-30

**Authors:** Maarten HT Zwartbol, Ina Rissanen, Rashid Ghaznawi, Jeroen de Bresser, Hugo J Kuijf, Kim Blom, Theo D Witkamp, Huiberdina L Koek, Geert Jan Biessels, Jeroen Hendrikse, Mirjam I Geerlings

**Affiliations:** 1 Department of Radiology, University Medical Center Utrecht and Utrecht University, Utrecht, the Netherlands; 2 Julius Center for Health Sciences and Primary Care, University Medical Center Utrecht and Utrecht University, Utrecht, the Netherlands; 3 Department of Radiology, Leiden University Medical Center, Leiden, the Netherlands; 4 Image Sciences Institute, University Medical Center Utrecht, Utrecht, the Netherlands; 5 Department of Geriatrics, University Medical Center Utrecht and Utrecht University, Utrecht, the Netherlands; 6 Department of Neurology, University Medical Center Utrecht and Utrecht University, Utrecht, the Netherlands

**Keywords:** Microinfarcts, cerebrovascular disease, cognitive functioning, dementia, cardiovascular risk factors

## Abstract

We determined the occurrence and association of cortical cerebral microinfarcts (CMIs) at 7 T MRI with risk factors, neuroimaging markers of small and large vessel disease, and cognitive functioning. Within the Medea-7T study, a diverse cohort of older persons with normal cognition, patients with vascular disease, and memory clinic patients, we included 386 participants (68 ± 9 years) with available 7 T and 1.5 T/3T brain MRI, and risk factor and neuropsychological data. CMIs were found in 10% of participants and were associated with older age (RR = 1.79 per +10 years, 95%CI 1.28–2.50), history of stroke or TIA (RR = 4.03, 95%CI 2.18–7.43), cortical infarcts (RR = 5.28, 95%CI 2.91–9.55), lacunes (RR = 5.66, 95%CI 2.85–11.27), cerebellar infarcts (RR = 2.73, 95%CI 1.27–5.84) and decreased cerebral blood flow (RR = 1.35 per −100 ml/min, 95%CI 1.00–1.83), after adjustment for age and sex. Furthermore, participants with >2 CMIs had 0.5 SD (95%CI 0.05–0.91) lower global cognitive performance, compared to participants without CMIs. Our results indicate that CMIs on 7 T MRI are observed in vascular and memory clinic patients with similar frequency, and are associated with older age, history of stroke or TIA, other brain infarcts, and poorer global cognitive functioning.

## Introduction

Cortical cerebral microinfarcts (CMIs), defined as microscopic regions of ischemic infarction in the cerebral cortex, have gained increasing attention because of their discovery on 7 tesla (7 T) magnetic resonance imaging (MRI).^1^,^2^ Previous studies have suggested that they are the most widespread type of brain infarction, often occurring in large numbers, and are correlated with measurable disruption of brain function.^3^ Furthermore, they may be a key part of the “silent” cerebrovascular burden, and have been associated with both small and large vessel disease.^2^,^3^

Frequency estimates of CMIs vary widely, likely depending on the study population, MRI field strength and rating criteria.^1–8^ Studies using 3 T MRI report estimates ranging from 6% in the general population, 20–29% in memory-clinic patients, to 57% in patients with cerebral amyloid angiopathy.^6–9^ At 7 T MRI, frequencies have been reported ranging from 27% to 72% in brains of non-demented persons, and from 55% to 86% in mild cognitive impairment (MCI) and Alzheimer’s disease patients.^1^,^4^,^5^

The current view regarding the etiology of CMIs is one of multiple causes. The main etiologies of CMIs are thought to be both cerebral small vessel disease (CSVD) and large vessel disease, the latter by means of hypoperfusion and microemboli.^2^ However, evidence to support these hypotheses is inconsistent, or scarce. Apart from studies in patients with cerebral amyloid angiopathy, associations with MRI features of CSVD are inconsistent.^6–8^,^10–15^ Studies regarding cerebral perfusion and CMIs are few.^16^ Only the microembolic etiology has substantial supporting evidence, by means of the association between carotid endarterectomy and acute CMIs.^17^ Furthermore, CMIs’ associations with risk factors such age, sex, smoking, alcohol use, hypertension, hyperlipidemia, and diabetes, are similarly inconsistent.^6–8^,^11–14^

In addition, CMIs have been associated with ante mortem cognitive decline and dementia in autopsy studies.^2^ A few neuroimaging studies have found similar associations in vivo.^11^,^12^,^14^

We think that more comprehensive studies in larger study populations are needed to elucidate risk factors, potential etiologies, associations with cognitive functioning, and clarify the importance of CMIs. Furthermore, studies at 7 T field strength are needed to get a more accurate prevalence of CMIs in vivo, since it detects almost four times the number of CMIs compared to 3 T.^11^

We utilized 7 T MRI to determine the frequency of CMIs in a diverse and large cohort of older persons with normal cognition, a history of vascular disease, or MCI or early Alzheimer’s disease. Furthermore, we examined associations between CMIs and vascular risk factors, neuroimaging markers of small and large vessel disease, and cognitive functioning.

## Materials and methods

### Study population

Data were used from the Memory Depression and Aging (Medea)-7T study, a cohort study at the University Medical Center (UMC) Utrecht, the Netherlands, with the objective to investigate risk factors and outcomes of brain changes defined on 7 T MRI. We recruited participants from four settings: 1) persons registered in one general practice in Utrecht, 2) participants of the PREDICT-MR study,^18^ 3) patients of the SMART-MR study,^19^ and 4) patients from the memory clinics of two hospitals in Utrecht. A detailed description of the recruitment settings is published elsewhere.^20^

1) Recruited general practice patients were ≥60 years; had no clinical diagnosis of mild cognitive impairment (MCI), dementia or other neurological conditions affecting cognition; had no terminal illness; had no previous medical evaluations for cognitive complaints; and had a Clinical Dementia Rating Scale (CDR) 0. 2) The PREDICT-MR study originated from a multicenter prospective cohort investigating major depressive disorder in adult primary care patients. Adult persons were recruited in waiting rooms of general practices, irrespective of the reason for consulting. 3) The SMART-MR study is a prospective cohort at the UMC Utrecht with the goal to investigate risk factors and clinical outcomes of MRI neuroimaging markers in patients with arterial disease. Adult persons newly referred to the UMC Utrecht for treatment of symptomatic atherosclerotic disease (coronary artery disease, cerebrovascular disease, peripheral arterial disease or abdominal aortic aneurysm) and without MRI contraindications were enrolled in the SMART-MR study. 4) Outpatient memory clinic patients were recruited from the UMC Utrecht and a general hospital in Utrecht if they had MCI or early Alzheimer’s disease (AD). Patients with moderate or severe AD were not included in the study. Inclusion criteria were age ≥60 years, a diagnosis of possible or probable AD according to the NINCDS-ADRDA workgroup criteria,^21^ or MCI according to Petersen criteria^22^; a CDR 0.5 or 1; and a Mini Mental State Examination score of ≥20.

A total of 368 participants were included in the Medea-7T study between January 2010 and October 2017: 70 from the general practice, 50 from the PREDICT-MR study, 213 from the SMART-MR study, and 35 from the memory clinics. All participants underwent a 7 T brain MRI using the same MRI sequences, and similar assessment of risk factors and outcomes and clinical examinations, all performed at the UMC Utrecht. Approval of the medical ethics committee of the UMC Utrecht was obtained according to the guidelines of the Declaration of Helsinki of 1975, and all participants provided written informed consent.

### 7T MRI protocol

A 7 T MRI scan of the brain was performed using a 7.0 T MRI system (Philips Healthcare, Cleveland, OH, USA) with a 16 or 32-channel receiver head coil (Nova Medical, Wilmington, MA, USA). The standard MRI protocol consisted of: a T1-weighted (3D acquisition; TI/TR/TE = 1225/4.8/2.2 ms; acquired voxel size = 1.00×1.00×1.00 mm^3^; reconstructed = 0.66×0.66× 0.50 mm^3^), T2-weighted Turbo-Spin Echo (3D acquisition; TR/TE = 3158/301 ms; acquired voxel size = 0.70×0.70×0.70 mm^3^; reconstructed = 0.35×0.35× 0.35 mm^3^), magnetization-prepared FLAIR (3D acquisition, TR/TE = 8000/300 ms, acquired voxel size = 0.80×0.80×0.80 mm³, reconstructed = 0.49×0.49× 0.49 mm³), and a dual echo susceptibility-weighted imaging (SWI) (TR/TE1/TE2 = 20/6.9/15.8 ms, acquired voxel size = 0.50×0.50×0.70 mm^3^, reconstructed = 0.40× 0.40×0.35 mm^3^) sequence.

### Assessment of cortical microinfarcts on 7 T MRI

Cortical CMIs were visually rated on 7 T MRI by one rater (MHTZ, 6 years of experience in neuroradiology), blinded to patient characteristics, on the T1-weighted, T2-weighted and FLAIR images according to proposed neuroimaging criteria.^2^ In brief, a CMI had to be a strictly intracortical lesion, visible in at least two directions, less than 4 mm in greatest dimension, hyperintense (to cortex) on T2-weighted imaging and hypointense on T1-weighted imaging. We did not use a FLAIR criterion since the FLAIR signal of a CMI ranges from hypo-, iso-, to hyperintense based on size and cavitation. Hence, we did not distinguish cavitated from non-cavitated lesions. A lesion was disregarded as a CMI if there was an accompanying susceptibility artefact on SWI, or if it was within 1 cm from a large cortical infarct. Size of a CMI was defined as the maximum diameter in any direction on T2-weighted imaging.

Intra-rater agreement was good with an intraclass correlation coefficient (20 cases, 2-way mixed-effects model, absolute agreement) of 0.85.

### Vascular risk factors

Questionnaires were used to assess age, sex, educational level, smoking, and alcohol use. Educational level, as a proxy of socioeconomic status, consisted of 8 levels, ranging from (un)completed primary school to academic degree, and was categorized into 4 categories in the analyses. Smoking was categorized as never, current, or former smoker. Alcohol use was expressed in units per week and categorized as none or <1 unit per week, 1–10 units per week, and >10 units per week. Systolic and diastolic blood pressures were both defined as an average of three measurements in supine position. Hypertension was defined as a mean systolic blood pressure of >140 mmHg or a mean diastolic blood pressure of >90 mmHg or use of antihypertensive drugs. Hypercholesterolemia was defined as a total cholesterol/high-density lipoprotein ratio ≥5.0 or use of lipid-lowering drugs. Weight (kg) and height (m) measurements without shoes and heavy clothing were used to calculated body mass index (BMI). Diabetes mellitus was defined as the use of antidiabetic medication, a known history of diabetes, a fasting glucose of ≥7.0 mmol/L or—in participants from PREDICT-MR—a non-fasting blood glucose ≥11.1 mmol/L.

### 1.5T and 3 T MRI protocol

In the PREDICT-MR and SMART-MR cohorts a 1.5 T whole-body system (Gyroscan ACS-NT, Philips Medical Systems, Best, the Netherlands) was performed in addition to the 7 T MRI. The standard MRI protocol consisted of a T1-weighted sequence (SMART-MR: 3D acquisition; repetition time (TR)/echo time (TE): 7.0/3.2 ms; voxel size = 0.94×0.94× 1.00 mm^3^ isotropic; PREDICT-MR: 3D acquisition; TR/TE: 6.9/3.1 ms; voxel size = 0.98×0.98×1.10 mm^3^ isotropic), a T2-weighted sequence (SMART-MR and PREDICT-MR: 2D acquisition; TR/TE 2200/10.5 ms; voxel size = 0.90×0.90×4.00 mm^3^), a FLAIR sequence (SMART-MR: 2D acquisition; TI/TR/TE 2000/6000/100 ms; 0.90×0.90×4.00 mm^3^; PREDICT-MR: 3D acquisition; TI/TR/TE 1600/4800/329.7 ms; voxel size = 0.98×0.98×1.10 mm^3^ isotropic), and a phase-contrast MR angiography sequence (SMART-MR and PREDICT-MR: 2D slice acquisition; TR/TE, 16/9 ms; voxel size = 0.98×0.98×5.00 mm^3^; velocity sensitivity 100 cm/s; acquisition at the level of the proximal cavernous segment of the ICA and prepontine basilar artery).

In the participants recruited from the general practice and the memory clinic patients, a 3.0 T MRI whole-body system (Philips Medical Systems, Best, the Netherlands) was used for brain imaging. The standard MRI protocol consisted of a T1-weighted sequence (3D acquisition; TR/TE = 8.0/4.5 ms; voxel size = 1.00×1.00×1.00 mm^3^ isotropic), a T2-weighted sequence (2D acquisition; TR/TE 3197.5/140 ms; 0.96×0.96×3.00 mm^3^), a FLAIR sequence (2D acquisition; TI/TR/TE 2800/11000/125 ms; voxel size = 096×0.96×3.00 mm^3^), and a phase-contrast MR angiography sequence (2D slice acquisition; TR/TE 14/8.8 ms; voxel size = 0.59×0.59×5.00 mm^3^; velocity sensitivity 100 cm/s; acquisition at the level of the proximal cavernous segment of the ICA and prepontine basilar artery).

### Assessment of neuroimaging markers of cerebrovascular disease

All assessments of cerebrovascular disease markers were performed blinded to patient characteristics. Cerebral infarcts and lacunes of presumed vascular origin^23^ were visually rated on 1.5 T or 3 T MRI by one rater (MHTZ), on the T1-weighted, T2-weighted and FLAIR images. Uncertain lesions were discussed during a consensus meeting between MHTZ and an experienced neuroradiologist (TDW) to reach agreement. Cerebral microbleeds were rated by one rater (MHTZ) on the 7 T MRI dual echo SWI images using a minimum intensity projection reconstruction and source data. Microbleeds were labeled as lobar or deep using the Microbleed Anatomical Rating Scale.^24^

Segmentation of gray matter, white matter, cerebrospinal fluid (CSF), and cortical thickness on 1.5 T or 3 T was performed using the Computational Anatomy Toolbox (Cat12; version 1155) using T1-weighted and FLAIR images.^25^ Segmentation of white matter hyperintensities (WMH) was performed using the lesion growth algorithm from the Lesion Segmentation Tool (LST; version 2.0.15; www.statistical-modeling.de/lst.html) using T1-weighted and FLAIR images, with a threshold of 0.1.^26^ Cat12 and LST are toolboxes implemented in the Statistical Parametric Mapping 12 (SPM12; version 6906; Wellcome Institute of Neurology, University College London, UK, http://www.fil.ion.ucl.ac.uk/spm/doc/) for Matlab (version 8.6; The MathWorks, Inc., Natick, Massachusetts, United States). Of note, we used lesion-filling on the T1-weighted sequence before segmenting it in Cat12, using the WMH segmentation from LST. Lesion-filling increases the T1-weighted signal of WMH and prevents incorrect labeling as gray matter. Furthermore, brain lesions were manually segmented on FLAIR images by a single rater (MHTZ) and used to correct the final (white/gray matter, CSF) Cat12 segmentations. Total brain volume (TBV; ml) was calculated by summing gray matter, white matter and total infarct volume. Intracranial volume (ICV; ml) was calculated by summing TBV and CSF.

Cerebral blood flow was calculated from the 1.5 T and 3 T phase-contrast MR angiography data using native Q-flow post-processing software on a stand-alone workstation (Philips, Best, the Netherlands). Blood flow through the prepontine basilar artery and the left and right cavernous internal carotid arteries was summed to calculate the total cerebral blood flow (ml/min). Of note, because flow was measured in the prepontine basilary artery, flow from proximal vertebrobasilar branches was not included in the measure of total CBF (e.g., posterior inferior cerebellar artery and pontomedullary perforators). Hence, the total CBF is a slight underestimation of actual CBF.

### Assessment of extracranial atherosclerosis

Markers of extracranial atherosclerosis were measured in the 213 participants of the SMART-MR cohort and included common carotid intima-media thickness (cIMT), carotid stenosis, and ankle-brachial index (ABI). An experienced technician performed carotid ultrasonography with a 10 MHz linear-array transducer. Mean cIMT was calculated from six measurements (anterolateral, posterolateral and mediolateral in both common carotid arteries). Carotid stenosis was assessed in the bilateral common and internal carotid arteries, and defined as the most severe stenosis, according to standard criteria based on the peak systolic velocity.^27^ ABI was calculated from the highest systolic blood pressure, measured by experienced technicians, at the posterior tibial and dorsal pedal arteries by Doppler and at both brachial arteries by a semiautomatic oscillometric device in supine position. We categorized carotid stenosis by presence of ≥50% stenosis, and ABI by ≤0.8, the clinical cutoff for peripheral artery disease.

### Neuropsychological assessment

The Mini Mental-State Examination was performed for all participants to compare the cognitive functioning characteristics of sub-cohorts. In addition, all patients underwent neuropsychological assessment for memory, executive functioning and working memory. Information processing speed was also assessed in the PREDICT-MR and SMART-MR cohorts. Memory was assessed with the 15 Word Learning Test, using a composite score of the immediate recall based on 5 trials, the delayed recall and the retention score; and the delayed recall of the Rey-Osterrieth Complex figure test.^28^,^29^ Executive functioning was assessed with the Verbal Fluency test using animals as categories (2 minutes).^30–32^ Working memory was assessed with the combined longest span scores of the Forward Digit Span and Backward Digit Span.^33^ Processing speed was assessed with the Digit Symbol Substitution Test (120 seconds).^34^ Composite Z-scores were calculated by converting raw scores to standardized Z-scores and averaging them across all subtests per domain. A global cognitive functioning composite Z-score was calculated by standardizing the cumulative of all averaged domain-specific Z-scores.

### Statistical analysis

We performed multiple imputation with 20 datasets to address missing values of studied risk factors, imaging markers of cerebrovascular disease, and cognitive functioning. Data were analyzed by pooling the 20 imputed datasets. Multiple imputation and statistical analysis were performed using SPSS Statistics for Windows, Version 25.0 (IBM, Armonk, NY, USA). Missing data ranged from 0.3% to 20%: level of education 0.5%; Mini-Mental State Examination 1.4%; BMI 0.3%; diabetes 0.8%; alcohol use 14.1%; infarcts 0.5%; cortical microinfarcts 3.0%; cerebral microbleeds 20%; intracranial volume 3.8%; total brain volume 3.8%; cortical thickness 3.8%; WMH volume 2.9%; and cerebral blood flow 2.7%.

Characteristics of vascular risk factors and MRI markers of cerebrovascular disease were calculated in the total study population and separate cohorts.

Modified Poisson regression with robust error variance was used to estimate relative risks (RR) for presence of CMIs, with vascular risk factors and MRI markers of cerebrovascular disease as independent variables, adjusted for age and sex (model 1) and additionally for educational level, history of stroke or TIA, BMI, smoking status, alcohol intake, hypertension, diabetes, and hypercholesterolemia (model 2). We used modified Poisson regression because it has similar flexibility and robustness as log-binomial regression but does not suffer from convergence errors. Furthermore, compared to log-binomial regression, it is less sensitive to omitted covariates.^35^ Analyses of TBV and WMH volume were also adjusted for ICV, and the analysis of CBF for TBV, in model 2. Moreover, the association with ≥50% carotid stenosis, cIMT and ABI was analyzed within the SMART-MR cohort (n = 213), using the same models.

ANCOVA was used to estimate age, sex and education level-adjusted mean cognitive functioning Z-scores, categorized by presence of no CMIs, 1–2 CMIs, and >2 CMIs. We used ANCOVA because of the low frequency and highly skewed distribution of CMIs. Furthermore, linear regression analysis was used to estimate the association between >2 CMIs and cognitive functioning (using no CMIs as reference category) and total number of CMIs and cognitive functioning and, with identical adjustments, in the total study population and separate cohorts. Residual plots of all linear regression analyses were checked for regression assumptions.

## Results

**Figure 1.**
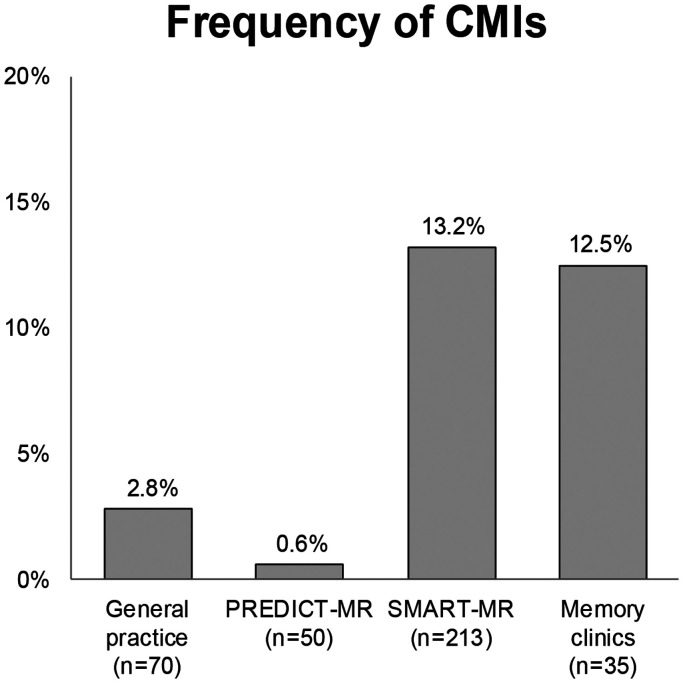
Presence of 1 or more cortical microinfarcts in each cohort.

Characteristics of the vascular risk factors and MRI markers of cerebrovascular disease in the 368 participants (68.3 ± 9.0 years; 30.4% women) are presented in Tables 1 and 2, respectively. A total of 129 CMIs were found in 10% (n = 35) of the study population, with a mean (min, max) number of 3.4 (1.0, 15.0) CMIs in affected brains. CMI frequencies per cohort ranged from 0.6% to 13.2% (Figure 1). Of the 58 participants with a history of stroke or TIA, 25.8% (n = 15) had CMIs. Within the SMART-MR cohort, 21% of the 62 participants with brain infarcts had CMIs. Within the memory clinic patients, 33% of the 6 participants with brain infarcts had CMIs.

**Table 1.** Characteristics of total study population and each included cohort in the Medea-7T study (N = 368).

	Total population (N = 368)	General practice (n = 70)	PREDICT-MR (n = 50)	SMART-MR (n = 213)	Memory clinics (n = 35)
Age (years)	68.3 ± 9.0	71 ± 7	60 ± 11	68 ± 8	75 ± 7
Women	30.4%	47%	62%	17%	31%
High educational level^a^	38.4%	66%	28%	33%	34%
Mini-Mental State Examination	28.6 ± 1.7	29.2 ± 1.2	28.9 ± 1.1	28.7 ± 1.5	26.4 ± 2.4
Body mass index (kg/m^2^)	26.6 ± 3.8	26 ± 4	26 ± 4	27 ± 4	25 ± 3
Smoking					
Never	23.6%	29%	48%	14%	37%
Former	63.0%	64%	40%	69%	60%
Current	13.3%	7%	12%	17%	3%
Alcohol					
No alcohol	13.4%	17%	18%	10%	23%
1–10 units p/week	60.1%	53%	72%	60%	57%
>10 units p/week	26.5%	30%	10%	30%	20%
History of stroke or TIA	15.8%	0%	0%	27%	3%
Hypertension	81.8%	73%	58%	90%	86%
Systolic BP	142 ± 18	148 ± 20	135 ± 16	140 ± 17	148 ± 18
Diastolic BP	80 ± 10	81 ± 10	80 ± 9	79 ± 10	79 ± 10
Diabetes	13.6%	3%	10%	17%	17%
Hypercholesterolemia	67.1%	23%	30%	92%	57%

Note: Values are presented as mean ± SD or percentages after imputation.

^a^Defined as higher vocational or academic degree.

BP: blood pressure.

**Table 2.** Cerebrovascular MRI markers in total study population and each included cohort in the Medea-7T study (N = 368).

	Total population (N = 368)	General practice (n = 70)	PREDICT-MR (n = 50)	SMART-MR (n = 213)	Memory clinics (n = 35)
Any infarct	20.7%	4%	6%	31%	17%
Cortical infarcts	6.3%	0%	4%	10%	3%
Lacunar infarcts	11.4%	1%	2%	18%	6%
Cerebellar infarcts	8.2%	1%	2%	12%	11%
Other infarcts	3.8%	1%	4%	4%	6%
Cortical microinfarcts	9.5%	3%	1%	13%	13%
Any microbleeds	61%	60%	49%	66%	45%
Lobar microbleeds	56%	51%	47%	62%	37%
Deep microbleeds	30%	26%	13%	37%	25%
Intracranial volume (ml)	1511 ± 142	1519 ± 151	1459 ± 154	1522 ± 135	1495 ± 139
Total brain volume (ml)	1111 ± 115	1103 ± 113	1099 ± 125	1128 ± 108	1040 ± 118
WMH volume (ml)^a^	3.4 (0.8, 15.2)	3.2 (0.9, 11.0)	2.5 (0.4, 11.2)	3.1 (0.7, 14.8)	6.8 (3.2, 27.3)
Cerebral blood flow (ml/min)	545 ± 136	580 ± 171	586 ± 140	527 ± 118	530 ± 139
Cortical thickness (mm)	2.54 ± 0.19	2.59 ± 0.08	2.55 ± 0.14	2.54 ± 0.15	2.49 ± 0.12

Note: Values are presented as mean ± SD or frequencies after imputation.

^a^Median (10th, 90th percentile).

WMH: white matter hyperintensities.

### Risk factors of CMIs

An increased RR of CMIs was found for older age (RR = 1.79 per +10 years, 95% CI 1.28 to 2.50) adjusted for sex; and for history of stroke or TIA (RR = 4.03, 95% CI 2.18 to 7.43) adjusted for age and sex (model 1, Table 3). These associations remained after further adjustments in model 2. Male sex was associated with increased RR of 3.08 (95% CI 1.07 to 8.83) for CMIs when adjusting for age and vascular risk factors in model 2. No association was found with educational level, BMI, smoking status, alcohol use, hypertension, diabetes or hypercholesterolemia and CMIs in our sample.

**Table 3.** Association between risk factors and presence of cortical microinfarcts.

	Cortical microinfarcts (yes vs. no) RR (95% CI)	
	Model 1	Model 2
Age, per +10 years	1.79 (1.28 to 2.50)	1.65 (1.04 to 2.61)
Male sex	2.66 (0.98 to 7.24)	3.08 (1.07 to 8.83)
Education, high vs. low-medium^a^	0.53 (0.16 to 1.77)	0.55 (0.14 to 2.25)
Body mass index, per +1 kg/m^2^	1.03 (0.94 to 1.12)	1.00 (0.91 to 1.09)
Smoking, current vs. never	1.34 (0.33 to 5.48)	1.28 (0.33 to 5.03)
Alcohol use		
No, or <1 unit/week	Reference	Reference
1–10 units/week	1.39 (0.45 to 4.36)	1.64 (0.00 to 4.62)
>10 units/week	1.42 (0.41 to 4.89)	1.28 (0.33 to 5.03)
History of stroke or TIA	4.03 (2.18 to 7.43)	4.15 (2.22 to 7.75)
Hypertension	2.36 (0.56 to 10.01)	2.54 (0.60 to 10.75)
Systolic BP, per +10 mmHg^b^	1.06 (0.87 to 1.28)	1.07 (0.90 to 1.27)
Diastolic BP, per +10 mmHg^b^	0.93 (0.65 to 1.33)	0.93 (0.67 to 1.29)
Diabetes	1.55 (0.68 to 3.50)	1.53 (0.65 to 3.61)
Hypercholesterolemia	1.31 (0.55 to 3.15)	0.83 (0.14 to 2.02)

Note: Values are relative risks (RR) with 95% confidence intervals calculated with modified-Poisson regression analysis with robust error variance.

Model 1: adjusted for age and sex. Model 2: model 1 with additional adjustment for educational level, history of stroke or TIA, body mass index, smoking status, alcohol use, hypertension, diabetes, hypercholesterolemia.

^a^High educational level indicates a higher vocational or academic degree (n = 142). Low-medium educational level indicates a lower vocational degree or lower (n = 122).

^b^In model 2 hypertension was replaced by systolic and/or diastolic blood pressure.

BP: blood pressure.

### Cerebrovascular disease on brain MRI and CMIs

An increased RR of CMIs was found for presence of all types of infarcts (Table 4). After adjusting for age and sex in model 1, the highest RR was observed for lacunes of presumed vascular origin (RR = 5.66, 95% CI 2.85 to 11.27) and cortical infarcts (RR = 5.28, 95% CI 2.91 to 9.55). The RR estimates decreased after adjustment for vascular risk factors in model 2, however, apart from cerebellar infarcts, confidence intervals did not cross 1. Decreased cerebral blood flow associated with an increased RR of 1.35 (95% CI 1.00 to 1.83) per -100 ml/min for CMIs in model 1. No associations with CMIs were found for microbleeds, total brain volume, WMH volume, or cortical thickness.

**Table 4.** Association between MRI markers of cerebrovascular disease and presence of cortical microinfarcts.

	Cortical microinfarcts (yes vs. no) RR (95% CI)	
	Model 1	Model 2
Any infarct, yes vs. no	5.34 (2.53 to 11.27)	3.96 (1.65 to 9.52)
Cortical infarct, yes vs. no	5.28 (2.91 to 9.55)	2.88 (1.23 to 6.73)
Lacunar infarct, yes vs. no	5.66 (2.85 to 11.27)	4.52 (2.17 to 9.38)
Cerebellar infarct, yes vs. no	2.73 (1.27 to 5.84)	1.73 (0.88 to 3.40)
Lobar microbleed, yes vs. no	1.22 (0.61 to 2.46)	1.15 (0.58 to 2.30)
Deep microbleed, yes vs. no	1.27 (0.57 to 2.81)	1.15 (0.52 to 2.55)
Total brain volume, per -10 ml^a^	1.02 (0.94 to 1.10)	0.99 (0.93 to 1.07)
Total WMH volume, per +1 natural-log (ml)^a^	1.00 (0.67 to 1.50)	0.91 (0.62 to 1.32)
Cortical thickness, per -0.01 mm	1.02 (0.99 to 1.05)	1.01 (0.98 to 1.04)
Cerebral blood flow, per -100 ml/min^b^	1.35 (1.00 to 1.83)	1.19 (0.92 to 1.55)

Values are relative risks (RR) with 95% confidence intervals calculated with modified-Poisson regression analysis with robust error variance.

Model 1: adjusted for age and sex. Model 2: model 1 with additional adjustment for educational level, history of stroke or TIA, body mass index, smoking status, alcohol use, hypertension, diabetes, hypercholesterolemia.

^a^Model 1 and 2 additionally adjusted for intracranial volume.

^b^Model 1 and 2 additionally adjusted for total brain volume.

WMH: white matter hyperintensities.

### Extracranial atherosclerosis and CMIs

Within the 213 participants (68.4 ± 8.2 years; 17.4% women) of the SMART-MR sub-cohort, increased RRs for presence of CMIs were found for higher cIMT (RR = 5.81 per +1 mm, 95% CI 2.55 to 13.21), ≥50% carotid stenosis (RR = 6.18, 95% CI 2.87 to 13.33), and ABI ≤ 0.8 (RR = 3.80, 95% CI 1.72 to 8.37). Additional adjustment for vascular risk factors in Model 2 did not materially change these associations (Table 5). Of the 19 persons with ≥50% carotid stenosis (ranging from ≥50-69% stenosis to occlusion), around 53% had presence of CMIs. Of the 149 persons with stenosis <50%, 10% were found to have CMIs. No CMIs were found in persons without carotid plaque or stenosis.

**Table 5.** Association between markers of extracranial atherosclerosis and presence of cortical microinfarcts in the SMART-MR cohort (n = 213).

	Cortical microinfarcts (yes vs. no) RR (95% CI)	
	Model 1	Model 2
Intima-media thickness, per +0.1 mm	5.81 (2.55 to 13.21)	4.66 (1.04 to 1.31)
Carotid stenosis ≥50% vs. <50%	6.18 (2.87 to 13.33)	6.33 (2.69 to 14.91)
Ankle-brachial index ≤0.8 vs. >0.8	3.80 (1.72 to 8.37)	4.24 (1.54 to 11.62)

Note: Values are relative risks (RR) with 95% confidence intervals calculated with modified-Poisson regression analysis with robust error variance.

Model 1: adjusted for age and sex. Model 2: model 1 with additional adjustment for educational level, history of stroke or TIA, body mass index, smoking status, alcohol use, hypertension, diabetes, hypercholesterolemia.

### CMIs and cognitive functioning

**Figure 2.**
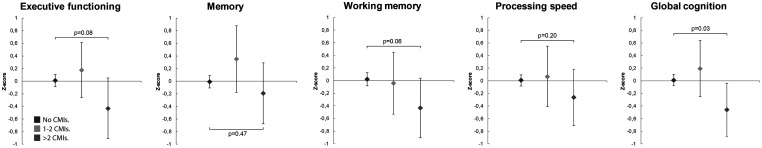
Cognitive functioning by presence of cortical microinfarcts. Values are estimated mean (95% CI) Z-scores, adjusted for age, sex and educational level. Executive functioning: no CMIs, 0.01 (−0.08 to 0.11); 1-2 CMIs, 0.18 (−0.26 to 0.61); >2 CMIs, −0.43 (−0.91 to 0.05). Memory: no CMIs, −0.01 (−0.11 to 0.09); 1-2 CMIs, 0.35 (−0.18 to 0.88); >2 CMIs, −0.19 (−0.68 to 0.29). Working memory: no CMIs, 0.02 (−0.08 to 0.13); 1-2 CMIs, −0.04 (−0.53 to 0.45); >2 CMIs, −0.43 (−0.90 to 0.04). Processing speed: no CMIs, 0.01 (−0.08 to 0.10); 1-2 CMIs, 0.07 (−0.41 to 0.55); >2 CMIs, −0.26 (−0.71 to 0.18). Global cognition: no CMIs, 0.01 (−0.08 to 0.10); 1-2 CMIs, 0.19 (−0.25 to 0.64); >2 CMIs, −0.46 (−0.88 to −0.04).

Figure 2 shows domain-specific and global cognitive functioning Z-scores adjusted for age, sex and educational level, according to presence of no CMIs (n = 333), 1 or 2 CMIs (n = 18) and more than 2 CMIs (n = 17). Participants with more than 2 CMIs had lower global cognitive functioning, compared to participants with no CMIs (mean difference = −0.48 standard deviations, 95% CI −0.91 to −0.05). Of all cognitive functions, presence of >2 CMIs seemed to be associated most with lower executive functioning (mean difference = −0.45 standard deviations, 95% CI −0.94 to 0.05) and working memory (mean difference = −0.46 standard deviations, 95% CI −0.95 to 0.02). Additional linear regression analyses showed a similar image. We found associations between CMIs and executive functioning (b = −0.08 per +1 CMI; 95% CI −0.16 to 0.00), working memory (b = −0.08 per +1 CMI; 95% CI −0.15 to −0.01), and global cognitive functioning (b = −0.08 per +1 CMI; 95% CI −0.15 to −0.02), with the latter being the strongest association. We did not observe an association with memory (b = −0.02 per +1 CMI; 95% CI −0.09 to 0.05) and processing speed (b = −0.05 per +1 CMI; 95% CI −0.13 to 0.03). The association between CMIs and cognitive functioning was similar in the SMART-MR and memory clinic cohorts as in the total sample (Supplement Table 1).

## Discussion

In this diverse cohort of 368 older adults with 7 T brain MRI we observed an overall CMI frequency of 10%, ranging from 1–3% in older persons with normal cognition, to 13% in patients with MCI or early AD, and 13% in patients with a history of vascular disease. Presence of CMIs was associated with older age, male sex, history of stroke or TIA, ischemic lesions on brain MRI and decreased cerebral blood flow. In a subcohort of persons with manifest arterial disease, presence of CMIs associated with extracranial atherosclerosis. Furthermore, presence of more than two CMIs was associated with lower global cognitive performance, in particular lower executive functioning and working memory.

Our study presents data on the largest 7 T brain MRI cohort to date and is one of the larger *in vivo* studies on CMIs currently published. Strengths of this study include 7 T MR imaging that is superior to lower field strength MRI for CMI detection,^11^ which should improve measures of CMI frequency and aid in the study of determinants. Furthermore, the inclusion of participants from various cohorts increased the variability in exposure and outcome status, allowed us to describe the occurrence of CMIs across these cohorts, and increases the generalizability of our results to other populations. Although it could be argued that combining different cohorts lead to incorrect estimates if effect modification by cohort was present, we have no a priori reason to assume that the relationships studied were different across cohorts. For instance, when we repeated the analyses with cognition within strata of cohorts, we found very similar effect estimates. A last strength is the extensive data on risk factors, neuroimaging markers and cognitive functioning.

Our study has several limitations. First, the cross-sectional design, which does not allow interpretation of causality. In particular, we cannot conclude which came first in the associations with brain MRI markers of small and large vessel disease and they may be explained by shared risk factors. Second, treatment of vascular risk factors before the 7 T MRI may have influenced associations with risk factors.^36^ Third, pooling cohorts with different in- and exclusion criteria can cause under- or overestimation of the examined associations. Fourth, CMIs and CMBs were rated on 7 T MRI data whereas the other MRI markers were evaluated on 1.5 T or 3 T MRI data. Ideally, all MRI markers should have been evaluated on 7 T MRI data because it provides a higher spatial resolution and contrast-to-noise ratio. However, it is currently unclear if STRIVE criteria are valid on 7 T MRI. Furthermore, as far as we know, there is no robust and validated brain segmentation software for 7 T MRI data. A related limitation are the differences in MR image quality between the various cohorts which may have caused variations in the accuracy of lesion rating and segmentation. However, apart from the PREDICT cohort, variations in image quality were visually minimal. In the PREDICT cohort the higher spatial resolution of the FLAIR sequence may have resulted in more accurate rating and segmentation of brain lesions, compared to the other cohorts. We tested for this by excluding the PREDICT cohort from the analyses, which caused no considerable change in associations (data not shown). Fifth, although the size of our population is large for a 7 T cohort, it may have had limited statistical power to detect small associations.

Previous neuroimaging studies with 3 T MRI have reported frequencies of CMIs ranging from 6% in non-demented older adults with hypertension,^6^ and a population-based cohort,^12^ 15% in acute stroke/TIA patients (also including acute CMIs)^7^ to 20–32% in memory clinic patients.^11^,^14^,^37^ Earlier 7 T studies reported much higher frequencies ranging from 27–72% in non-demented elders^1^,^38^ to 55–86% in MCI/AD patients.^4^,^5^ The frequencies observed in this 7 T study are much lower, which might be explained by the evolving insight regarding neuroimaging characteristics of CMIs and changes in imaging criteria,^2^ which may have increased the diagnostic accuracy leading to a decreased number of detections. Furthermore, the relatively low CMI frequency in our memory clinic population, compared to prior studies, might be explained by the comparatively lower overall cerebrovascular disease burden compared to previous studies.

With regard to vascular risk factors, older age, male sex, and prior stroke or TIA were associated with presence of CMIs in this study. An association with prior stroke or TIA is the most consistently reported association in previous literature.^7^,^11^,^12^,^14^ Associations with vascular risk factors in prior reports are conflicting, with not one conventional risk factor showing a consistent relationship. Age has been associated with CMIs in some studies,^6^,^12^ but not in others.^7^,^8^,^14^,^37^ Male sex shows a similar picture, being associated with CMIs in some studies^7^,^11^,^14^ and absent in others.^6^,^8^,^12^,^37^ All other risk factors show equivocal associations across neuroimaging and neuropathology studies, which was noted in a recent review article on CMIs.^2^

In regard to imaging markers of cerebrovascular disease, we found that all types of brain infarcts on MRI were associated with presence of CMIs, which is in line with prior neuroimaging,^12^,^14^,^37^ and neuropathology studies.^39–41^ Apart from lacunes of presumed vascular origin, we did not find associations with CSVD markers such as WMH, microbleeds or brain atrophy. Associations of CMIs with WMH have been found in several studies,^12–14^ but were absent in others.^6–8^,^37^ Associations with cerebral microbleeds were similarly inconsistent,^7^,^8^,^11^,^12^,^14^ as are the few results for brain atrophy.^8^,^11^,^37^ An explanation for these inconsistencies could be that most evidence is based on studies with small to modest sample sizes, studies conducted in different settings and study populations, examining different determinants and neuroimaging markers. In this study we used accurate and comprehensive methods to assess CMIs and their risk factors, potential etiologies, and cognitive consequences in a large and generalizable study population to increase the consistency of evidence.

We observed strong associations between markers of extracranial atherosclerosis and presence of CMIs in a subcohort of persons with manifest arterial disease. These results are in concordance with prior studies, although associations of CMIs with cIMT and ABI have not been previously studied. These associations are likely a reflection of the systemic nature of atherosclerosis that is in line with the well-established association between CMIs and intracranial atherosclerosis.^12^,^37^,^42^,^43^ Furthermore, we observed an association between lower cerebral blood flow and presence of CMIs, which is in line with a recent study in memory clinic patients.^16^ Lower CBF is regarded a proxy of cerebral hypoperfusion, one of the proposed etiologies of CMIs.^2^ However, CBF can be low due to a problem with supply or due to decreased metabolic demand from diseased or injured tissue.^44^ Since we do not have data on cerebral metabolism, we cannot make this distinction. Hence, the current finding may also represent decreased metabolic demand. Overall, CMIs showed consistent associations with markers of large arterial disease and not with specific small vessel disease lesions. Although the association with lacunes might be regarded as discrepant, lacunes are caused by small and large vessel disease.^45^

Concerning the cognitive outcomes of CMIs, we observed that presence of more than 2 CMIs was associated with lower cognitive performance, most evident for global cognitive functioning. Our findings suggest that especially executive functioning and working memory might be affected due to high CMI burden. These findings are in concordance with most *in vivo* studies.^7^,^11^,^12^,^14^ The small number of participants with 1 and more than 2 CMIs might have unpowered the analyses of specific cognitive domains, nonetheless, the estimated effect sizes were similar to prior studies.^11^,^12^,^14^ Our findings suggest that CMIs are one of the pathways through which large vessel disease could contribute to cognitive decline, which is potentially modifiable.

In conclusion, CMIs on 7 T MRI were observed in vascular and memory clinic patients with similar frequency, but they were less frequent than reported in previous studies. Our results suggest that CMIs are a manifestation of the severity of systemic arterial disease, possibly through the pathways of hypoperfusion and microemboli. In addition, presence of more than two CMIs was associated with significantly poorer global cognitive functioning, suggesting that CMIs could be an important vascular contributor to cognitive decline.

## Supplemental Material

sj-pdf-1-jcb-10.1177_0271678X211025447 - Supplemental material for Cortical cerebral microinfarcts on 7T MRI: Risk factors, neuroimaging correlates and cognitive functioning – The Medea-7T study Click here for additional data file. Supplemental material, sj-pdf-1-jcb-10.1177_0271678X211025447 for Cortical cerebral microinfarcts on 7T MRI: Risk factors, neuroimaging correlates and cognitive functioning – The Medea-7T study by Maarten HT Zwartbol, Ina Rissanen, Rashid Ghaznawi, Jeroen de Bresser, Hugo J Kuijf, Kim Blom, Theo D Witkamp, Huiberdina L Koek, Geert Jan Biessels, Jeroen Hendrikse and Mirjam I Geerlings in Journal of Cerebral Blood Flow & Metabolism
