# Supplementary material for: Cortical cerebral microinfarcts on 7T MRI: Risk factors, neuroimaging correlates and cognitive functioning – The Medea-7T study
Source: J Cereb Blood Flow Metab. 2021 Jun 30;41(11):3127–38. doi: 10.1177/0271678X211025447 (PMC8543666; doi:10.1177/0271678X211025447)
Supplement: sj-pdf-1-jcb-10.1177_0271678X211025447 - Supplemental material for Cortical cerebral microinfarcts on 7T MRI: Risk factors, neuroimaging correlates and cognitive functioning – The Medea-7T study [file sj-pdf-1-jcb-10.1177_0271678X211025447.pdf]

Supplement Table 1. Association between CMIs and cognitive functioning in subcohorts

	Medea-7T (N=368)	SMART-MR (n=213)	Memory clinics (n=35)	General practice (n=70)	PREDICT-MR (n=50)
CMIs (%)	9.5%	13%	13%	3%	1%
Global cognition (z-score)[a]	0±1	-0.04±1.00	-1.15±1.11	0.34±1.04	0.48±0.93
Executive functioning (z-score)[a]	0±1	-0.06±1.19	-0.75±1.44	0.30±1.70	0.36±1.00
Working memory (z-score)[a]	0±1	-0.02±1.00	-0.42±0.88	0.25±1.08	0.04±0.87
CMIs vs. global cognitive functioning (b, (95% CI))[b]	-0.08 (-0.15 to -0.01)	-0.07 (-0.15 to 0.01)	-0.06 (-0.22 to 0.10)	N/A	N/A
CMIs vs. executive functioning (b, (95% CI))[b]	-0.08 (-0.16 to 0.00)	-0.08 (-0.16 to 0.01)	-0.05 (-0.25 to 0.15)	N/A	N/A
CMIs vs. working memory (b, (95% CI))[b]	-0.08 (-0.15 to-0.01)	-0.08 (-0.17 to 0.02)	-0.08 (-0.22 to 0.07)	N/A	N/A

[a] presented as mean ± standard deviation.

[b] linear regression analysis with number of cortical cerebral microinfarcts (CMIs) as independent variable and cognitive domain as dependent variable, adjusted for age, sex and educational level.

N/A. No valid analyses possible due to low frequency of CMIs.
